# Pharmacological Treatment of Cognitive Impairment Associated With Schizophrenia: State of the Art and Future Perspectives

**DOI:** 10.1093/schizbullopen/sgae013

**Published:** 2024-05-23

**Authors:** Antonio Vita, Gabriele Nibbio, Stefano Barlati

**Affiliations:** Department of Clinical and Experimental Sciences, University of Brescia, Brescia, Italy; Department of Mental Health and Addiction Services, ASST Spedali Civili of Brescia, Brescia, Italy; Department of Clinical and Experimental Sciences, University of Brescia, Brescia, Italy; Department of Clinical and Experimental Sciences, University of Brescia, Brescia, Italy; Department of Mental Health and Addiction Services, ASST Spedali Civili of Brescia, Brescia, Italy

**Keywords:** antipsychotics, drugs, cognition, schizophrenia, pharmacological treatment

## Abstract

Cognitive Impairment Associated with Schizophrenia (CIAS) represents one of the core dimensions of Schizophrenia Spectrum Disorders (SSD), with an important negative impact on real-world functional outcomes of people living with SSD. Treatment of CIAS represents a therapeutic goal of considerable importance, and while cognition-oriented evidence-based psychosocial interventions are available, effective pharmacological treatment could represent a game-changer in the lives of people with SSD. The present critical review reports and discusses the evidence regarding the effects of several pharmacological agents that are available in clinical practice or are under study, commenting on both current and future perspectives of CIAS treatment. In particular, the effects on CIAS of antipsychotic medications, anticholinergic medications, benzodiazepines, which are currently commonly used in the treatment of SSD, and of iclepertin, d-serine, luvadaxistat, xanomeline-trospium, ulotaront, anti-inflammatory molecules, and oxytocin, which are undergoing regulatory trials or can be considered as experimental agents, will be reported and discussed. Currently, available pharmacological agents do not appear to provide substantial benefits on CIAS, but accurate management of antipsychotic medications and avoiding treatments that can further exacerbate CIAS represent important strategies. Some molecules that are currently being investigated in Phase 2 and Phase 3 trials have provided very promising preliminary results, but more information is currently required to assess their effectiveness in real-world contexts and to provide clear recommendations regarding their use in clinical practice. The results of ongoing and future studies will reveal whether any of these molecules represents the awaited pharmacological game-changer in the treatment of CIAS.

**Key Points**

- Second-generation antipsychotics (SGAs) > first-generation antipsychotics (FGAs), but no clear superiority of a single SGA.
- Third-generation antipsychotics (TGAs) appears to be promising.
- Avoid prolonged and high-dose use of anticholinergics and benzodiazepines (BDZs).
- Memantine and galantamine appear to be promising.
- Other available add-on molecules are not consistently useful.
- Newer molecules are promising, particularly iclepertin, luvadaxistat and xanomeline-trospium.
- *N*-acetylcysteine and mynocicline appear to be promising among anti-inflammatory molecules.

## Introduction

Schizophrenia Spectrum Disorders (SSD) represent severe and often debilitating mental disorders that frequently have a consistent negative impact on people’s functioning and real-world outcomes.^1–4^

Cognitive deficits represent one of the core features of SSD^5,6^ as well as one of the main determinants of functional impairment in people living with SSD.^7–10^ This issue has such a clinical relevance that the international scientific community is currently leaning toward to use a dedicated term, that of Cognitive Impairment Associated with Schizophrenia (CIAS), to describe this phenomenon.^11^ From the perspective of improving the global functioning and real-world outcomes of people living with SSD, CIAS represents a fundamental treatment target.^6,12–14^

Currently, available antipsychotic medications are consistently effective in improving psychotic symptoms^15,16^ and even in reducing long-term mortality,^17–19^ but their impact on other core domains of SSD such as primary negative symptoms^20,21^ and CIAS^22,23^ remains more limited.

To date, the treatments that appear to provide the more consistent benefits on CIAS are evidence-based psychosocial interventions such as cognitive remediation^24–27^ and physical exercise-based programs.^28–30^

However, an appropriate management of antipsychotic therapy as well as of other pharmacological agents routinely used in the treatment of SSD can have a determinant impact on CIAS.^14^ Moreover, several pharmacological agents with the potential of providing significant improvements in CIAS have been developed in recent years,^31–33^ and their effects in controlled clinical trials and real-world studies, both alone and combined with stable antipsychotic treatment, are currently the object of investigation and of great scientific interest, with some preliminary findings already emerging.

The aim of the present critical review is to provide a detailed overview of the available evidence regarding the effects of currently available pharmacological treatments and of novel pharmacological agents for the treatment of CIAS.

## ANTIPSYCHOTIC MEDICATIONS

Antipsychotic medications represent the cornerstone of the treatment of SSD and are essential to obtain psychotic symptoms remission and clinical stabilization,^16,34^ which represent the first steps of the process of recovery.^35–37^

Several systematic reviews and meta-analyses have explored the effects of antipsychotic treatment on CIAS: second-generation antipsychotic medications appear to provide small benefits and have been shown to have substantially superior cognitive outcomes compared to first-generation molecules, which are globally outperformed by placebo.^22,38,39^ However, no substantial differences have been observed between different second-generation molecules.^23,40^ Long-acting injectable formulations, while outperforming oral formulations on important outcomes such as risk of hospitalizations and relapse, do not appear to provide superior effects on CIAS.^41^

Recently developed second-generation antipsychotics such as lurasidone, cariprazine, brexpiprazole, and lumateperone could have a favorable cognitive profile compared to other molecules, but the evidence in this regard is still preliminary and requires further research.^42–45^ In particular, antipsychotics that interact as partial dopamine receptor agonists rather than acting as full antagonists, such as aripipraxole, cariprazine, brexpiprazole, and lumateperone, sometimes defined “third generation antipsychotics,”^46,47^ have been postulated to have a particularly favorable cognitive profile^48^; however, more direct evidence of their positive effects on CIAS has to be observed to provide clear treatment recommendations.

Clozapine may not provide substantial cognitive benefits compared to other antipsychotic molecules in the whole SSD population,^22^ but it could provide some measure of improvement also on CIAS in an individual with Treatment-Resistant Schizophrenia (TRS).^49^ In fact, a recent meta-analysis including 50 studies focusing on CIAS effects of clozapine in TRS observed a significant positive effect on global cognitive measures, albeit of minimal size (Standardized Mean Difference = 0.11).^50^

Of note, a large systematic review, pairwise, and network meta-analysis exploring the effects of antipsychotic medications on cognitive performance is currently underway^51^: this work aims to both incorporate the most recent evidence and maintain very high methodological quality standards and could provide valuable insight on the relationship between antipsychotic therapy and CIAS treatment.

## ANTICHOLINERGICS AND BENZODIAZEPINES

Recent evidence shows that the total anticholinergic burden in people with SSD is directly related to increased severity of CIAS,^52–54^ resulting in a reduction of functional capacity,^55,56^ and of real-world functional outcomes.^57^ According to some studies, the total anticholinergic burden also appears to directly limit the effectiveness of psychosocial interventions aimed at improving CIAS,^58,59^ while another study suggests that cognitive remediation might mitigate the negative effects of anticholinergic burden on CIAS.^60^

Overall, the use of anticholinergic medications should be kept to a minimum and used only when strictly necessary in people living with SSD. Furthermore, the anticholinergic component of antipsychotic medications should also be taken into account while optimizing long-term therapy, particularly in individuals showing significant CIAS.

Benzodiazepines represent another category of medications that are frequently prescribed in people living with SSD that appear to have a consistent negative impact on CIAS,^61^ particularly in older patients,^62^ and indeed they are considered a well-known risk factor for cognitive decline in elderly subjects.^63–65^

Again, the use of benzodiazepines, while essential in some instances, should be kept to a minimum in people living with SSD, particularly in elderly patients, and should not be maintained for prolonged periods of time.

## ADJUNCTIVE AGENTS TO ANTIPSYCHOTICS IN THE TREATMENT OF CIAS

Several molecules that are already available and used to treat different conditions have been considered as potential add-ons to antipsychotic treatment specifically with the purpose of improving CIAS.^13,33,66,67^

Antidepressant medications have been explored as a potential treatment for CIAS in several studies, but recent systematic reviews and meta-analyses have found minimal effect both considering the pharmacological category in its entirety and considering individual molecules.^66,68–70^

Ondansetron, 5-HT3 receptor antagonist that is commonly used to treat nausea and vomiting, particularly in oncological conditions, has been investigated in several trials but provided inconsistent results.^71^

Buspirone, which interacts with presynaptic 5-HT1A autoreceptors and is mainly used to treat anxiety, has been reported to provide small benefits in some trials, but results are again inconsistent.^72,73^

Raloxifene, a selective estrogen modulator that is commonly used in the treatment of postmenopausal osteoporosis and in some cases in oncological conditions such as breast cancer, has been hypothesized to also exert an effect in the central nervous system, but no substantial positive effect on CIAS has been reported.^74–77^

Metformin, a biguanide oral antidiabetic that is commonly used also for metabolic control in overweight individuals, has been recently investigated in a small trial showing promising positive effects on CIAS that require further replication.^78^

Acetylcholinesterase inhibitors that are used in the initial stages of neurodegenerative conditions producing cognitive impairment and dementia have been investigated, in different meta-analyses: moderate positive effects were observed in the processing speed domain and small effect on the attention domain, but no effect on global cognitive performance or other cognitive domains was reported.^79,80^

Memantine in particular appeared to produce the most significant improvements. More recent randomized clinical trials have explored the effects of add-on memantine in the treatment of CIAS: significant positive effects were observed in several cognitive domains measured with different neuropsychological tests,^81–83^ and one study also reported enhanced auditory discrimination learning.^84^ These results require consistent reproduction in larger samples, but memantine currently represents the molecule with the most promising evidence among those that are commercially available in the present moment.

Another molecule that showed positive effects on CIAS is galantamine. Galantamine acts as a reversible, competitive acetylcholinesterase inhibitor but also as a positive allosteric modulator of alpha-7 nicotinic acetylcholine receptors and it is currently approved for the treatment of Alzheimer’s Disease, where it shows consistent positive effects in individuals with mild or moderate cognitive impairment.^85,86^ A systematic review and meta-analysis investigating its effects in people living with SSD, including 6 trials and a total of 226 participants, reported no significant effect on positive and negative symptoms, but a significant small-sized effect on CIAS.^87^ While these effects have to be reproduced on a larger scale to draw clear conclusions, they appear promising, and it can be hypothesized that a combination of medications that each provide small benefits could produce clinically meaningful improvements.^88^

## NEW DRUGS IN DEVELOPMENT FOR THE TREATMENT OF CIAS

Imbalances in neurotransmitter systems, including the glutamatergic, GABAergic, and cholinergic systems, may all contribute to the development and severity of CIAS. For instance, the glutamatergic system is heavily involved in the transmission of excitatory signals, and it has been hypothesized that positively modulating glutamatergic function could have positive effects on CIAS.^89–92^ The GABAergic system, which utilizes gamma-aminobutyric acid (GABA) as its main inhibitory neurotransmitter, and the cholinergic system, revolving around the acetylcholine neurotransmitter, are involved in the regulation of several cognitive processes and could represent treatment targets to address CIAS.^93–96^

Moreover, inflammation has also been suggested to play a relevant role in the pathophysiology of SSD, and persisting inflammatory processes may consistently contribute to CIAS.^97–104^

All these systems represent interesting targets related to cognitive performance, and several different molecules interacting with them in different ways are being investigated or are in the process of being developed for the treatment of CIAS.

## ICLEPERTIN

Iclepertin (BI 425809) is a selective glycine transporter 1 (GlyT1) inhibitor that has been developed and is currently being investigated specifically as an add-on to antipsychotic therapy for the treatment of CIAS.^105^ GlyT1, which is expressed in glial cells and at a presynaptic level in neurons, regulates the reuptake and release of glycine, which in turn acts as a *N*-methyl-d-aspartate (NMDA) receptor agonist.^106,107^ NMDA receptor activity is crucial for glutamatergic neurotransmission, as well as for glutamatergic network excitatory/inhibitory balance and synaptic plasticity, which play a key role in cognitive functioning.^89,90^

Preclinical studies in rodent models have shown positive effects on cognitive outcomes,^108,109^ while several Phase I (NCT02068690, NCT02337283, NCT02362516, NCT02383888) studies have attested the safety and the pharmacokinetic profile of the molecule: drug-related adverse effects typically appear with higher dosages, are generally mild and include headache, somnolence, fatigue, vertigo, and blurred vision.^110–112^

A Phase II study (NCT02832037) showed very promising results: 509 participants were randomly allocated to 2, 5, 10, and 25 mg dose groups of add-on iclepertin or placebo with a 1:1:1:1:2 proportion and assessed with the MATRICS Consensus Cognitive Battery (MCCB)^113^ and the Schizophrenia Cognition Rating Scale (SCoRS).^114^ Significant group-related effects were observed at the study endpoint of 12 weeks, with the 10 and the 25 mg dose groups outperforming placebo on the MCCB global cognition score. A significant dose-response effect was also observed, although the 10 and 25 mg doses did not produce significantly different effects.^115^

However, no significant treatment-related effect was observed as regards SCoRS scores. Moreover, electroencephalography data were recorded from a subgroup of patients (*n* = 79) at baseline and end of treatment, measuring mismatch negativity, auditory steady-state response, and resting state gamma power as correlates of response, and no significant treatment-related effect was observed.^116^

Another Phase II study (NCT03859973) compared iclepertin 10 mg combined with computerized cognitive training (CCT) to CCT combined with placebo, randomizing with a 1:1 proportion of a total of 200 participants.^117^ The results of the study were recently posted on clinicaltrials.gov, and the combination of iclepertin and CCT did not appear superior to CCT and placebo on MCCB and SCoRS scores at the 12-week endpoint.

Three Phase III trials investigating the effects of iclepertin 10 mg, CONNEX 1 (NCT04846868), CONNEX 2 (NCT04846881), and CONNEX 3 (NCT04860830), as well as an additional open-label safety extension trial, CONNEX-X, are currently underway and will provide additional and valuable information to better understand the potential usefulness of this molecule.

## d-SERINE

d-Amino acids such as d-serine and d-aspartate represent agonists and activators of NMDA receptors, again increasing the activity of glutamatergic pathways and potentially improving CIAS.^118^d-Serine in particular has been investigated as a potential add-on treatment for CIAS.^119^ One open-label trial investigated the effect of 30, 60, or 120 mg/kg d-serine in a sample of 42 SSD participants and observed a significant positive effect on the MCCB global cognition scores for doses of 60 and 120 mg/kg.^120^ However, one study compared a 18-week trial of 2 g d-serine to a placebo in a sample of 195 participants, observing no significant effect on MCCB scores.^121^ Another study included 104 participants and 4 treatment arms: d-serine 30 mg/kg and CCT, d-serine and active control to CCT, CCT and placebo, active control to CCT and placebo: the augmentation with d-serine did not provide superior cognitive effects.^122^ Despite the potential positive effect of d-serine on NMDA receptors, it should be considered that d-serine is rapidly metabolized in the human body, potentially requiring high doses to produce noticeable effects and consideringly limiting its effectiveness as a pharmacological agent.^123^

## LUVADAXISTAT

Inhibiting the activity of d-amino acid oxidase (DAAO) could represent an effective pathway to increase d-amino acid effects on NMDA receptors.^124,125^

A recent systematic review and meta-analysis investigated the effects of sodium benzoate, a molecule acting as a DAAO inhibitor, on several core SSD outcomes: while significant positive effects were observed for positive symptoms, no significant effect was observed for other outcomes, including CIAS (4 studies included in the meta-analysis on global cognition).^126^

Luvadaxistat (TAK-831 and NBI-1065844) acts as a selective and potent DAAO inhibitor and has shown positive effects on neurocognitive and social cognition outcomes in rodent models of SSD.^127^

As regards human trials, luvadaxistat effects as an add-on treatment of CIAS were assessed using the Brief Assessment of Cognition in Schizophrenia (BACS)^128^ in two complete Phase II trials. In one trial (NCT03359785), investigating luvadaxistat 50 and 500 mg compared to placebo for 8 days in 31 participants, significant effects were observed on mismatch negativity with 50 mg, but not with 500 mg,^129^ while analyses on BACS data are currently undergoing quality control procedures by the National Library of Medicine as reported on clinicaltrials.gov.

In the other trial, the INTERACT study (NCT03382639) luvadaxistat 50, 125, and 500 mg were compared with a placebo in a sample of 315 participants. The results, reported on clinicaltrials.gov, show no significant effect on the main study outcomes, which was negative symptoms severity, but appear promising as regards BACS scores; again, BACS data analyses data are currently undergoing quality control procedures by the National Library of Medicine. In both trials, luvadaxistat showed a good tolerability profile, with one patient reporting dyspepsia in NCT03359785 and 7 patients reporting headache (5 at 125 mg dose and 2 at 500 mg dose) in the INTERACT study.

The ERUDITE Phase II trial (NCT05182476) is currently undergoing recruitment and aims to recruit a total of 200 participants. It will compare luvadaxistat and placebo and consider CIAS measured with the BACS as the primary outcome: results from this study will provide more definitive information on the potential effectiveness of this molecule on CIAS.

## XANOMELINE-TROSPIUM

Xanomeline is a muscarinic agonist with a selective profile for the M1, M4, and M5 acetylcholine receptors.^130^ Doses are required to exert significant effects in the central nervous systems, however, frequently produce significant peripheral adverse effects on the gastrointestinal tract such as nausea, vomiting, and diarrhea as well as other cholinergic activation manifestations such as sweating, excessive salivation, and syncope. Trospium is a peripheral muscarinic receptor antagonist that does not cross the blood–brain barrier and therefore represents an effective strategy to reduce xanomeline-induced cholinergic adverse effects without hindering its positive effects at a central nervous system level.^131,132^ Xanomleine-trospium (KarXT) efficacy on core SSD symptoms including CIAS was explored in the EMERGENT-1 Phase 2 trial (NCT03697252). The trial included 125 participants, 60 allocated to receive from 50/20 to 125/30 mg twice daily of xanomeline-trospium and 65 to placebo, that were assessed, among other measures, with the computerized Cogstate Brief Battery (CBB) to measure CIAS. While the primary analysis did not reveal significant treatment-related effects on CIAS, post hoc analyses conducted removing outliers emerged as significant. Moreover, if the analysis was limited to participants showing significant CIAS, measured as a composite CBB *Z*-score < −1, the observed effect was not only significant but also of moderate effect size (*d* = 0.50). Finally, CIAS improvement was not related to SSD symptom severity changes.^133^ The results of the EMERGENT-2 Phase 3 trial (NCT04659161) were also recently published: this trial included 252 participants, 126 allocated to receive xanomeline-trospium and 125 to receive placebo, and showed a significant positive effect on SSD symptoms severity.^134^ However, results on CIAS were not disclosed as a part of this trial.

In both trials, reported side effects were mostly of mild severity and were related to the cholinergic effects of xanomeline, including gastrointestinal disorders (such as constipation, nausea, dry mouth, dyspepsia, and vomiting), headache, and akathisia. As regards serious adverse effects, 2 participants reported suicidal ideation in the EMRGENT-2 trial.

The EMERGENT-3 (NCT04738123) and the EMERGENT-4 (NCT04659174) Phase 3 trials have been completed, but results are still to be disclosed, while the EMERGENT-5 (NCT04820309) trial and a trial assessing the effects of xanomeline-trospium as add-on treatment to other antipsychotic therapy (NCT05145413) are still underway. Xanomleine-trospium is also currently being investigated as a treatment for Alzheimer-related psychosis in two trials (NCT05511363 and NCT06126224).

## ULOTARONT

Ulotaront (SEP-363856) acts as a trace amine-associated receptor 1 (TAAR1) agonist: TAAR1 is a G-protein coupled receptor expressed in neurons located in monoaminergic nuclei that has been shown to affect dopaminergic, serotonergic, and glutamatergic signaling and is hypothesized to impact affective dimensions and cognitive processes in SSD as well as in other disorders.^135,136^ Preliminary evidence of the efficacy of ulotaront, mainly as an antipsychotic, was shown in Phase 2 trials (NCT02969382) and open-label extensions (NCT02970929).^137–139^ However, two major Phase 3 studies did not observe a significant positive effect of ulotaront when compared to placebo: the DIAMOND-1 (NCT04072354) study included a total of 435 participants and evaluated 50 mg and 75 mg dosages and the DIAMOND-2 (NCT04092686) included a total of 464 participants and evaluated 75 mg and 100 mg dosages. These negative findings have been related also to the observation of a large placebo effect and to the difficulties in the trials conduction due to the COVID-19 pandemic,^140^ but have discouraged further exploration of this molecule as a valid treatment for CIAS.

## ANTI-INFLAMMATORY MOLECULES, NEUROSTEROIDS, AND IMMUNOMODULATORS

Inflammatory processes involving central nervous system structures and glial systems, cytokines alteration, and neurosteroid imbalances represent pathophysiological characteristics of SSD that could have a persistent and long-standing negative effect on several core symptom domains, including CIAS.^97,99,100,102–104^ In this regard, several systematic reviews and meta-analyses have investigated the effects of anti-inflammatory molecules, neurosteroids, and immunomodulators in the treatment of SSD.^141–143^

One meta-analysis included CIAS as a treatment outcome: 14 studies on aspirin, omega-3, estrogen, selective estrogen receptor modulators, davunetide, minocycline, pregnenolone, and erythropoietin were included in the analyses, reporting significant but small-sized positive effects on neurocognitive performance for both minocycline (*k* = 10) and pregnenolone (*k* = 20) add-on treatments.^144^

Another meta-analysis did consider CIAS as a treatment outcome, including 18 studies, but concluded that the excessive heterogeneity of CIAS assessment measures did not allow a qualitative synthesis; however, they commented that a positive effect on CIAS was reported for minocycline, davunetide an *N*-acetylcysteine in some studies.^145^

Another meta-analysis included a quantitative synthesis combining the effects of several anti-inflammatory molecules and separately considered the different neurocognitive domains: a significant small-sized effect was observed for working memory (17 studies), but no effect was observed for neurocognitive composite scores (12 studies) or the other cognitive domains.^146^

*N*-acetylcysteine appears as a particularly promising molecule, as it appears to have both global anti-inflammatory and antioxidant effects and a positive interaction with NMDA receptors.^147^ A meta-analysis of trials on the effects of *N*-acetylcysteine in SSD included CIAS as an outcome: overall composite scores were not calculated by the Authors, and a quantitative synthesis of global cognition effects was not performed as only one included study reported this specific outcome. Results regarding the working memory domain (3 trials) showed a significant, moderate-sized (Standardized Mean Difference = 0.56) effect, while those on the processing speed domain (3 trials) showed a small-sized (Standardized Mean Difference = 0.27) non-significant effect.^148^

Minocycline is another molecule of substantial interest, as it has shown small but significant positive effects both in systematic and meta-analytic investigations^144,145^ and in recent trials,^149^ but more recent trials reporting negative results have also been published.^150^

While these results can be considered as promising, more research is currently needed to better establish the potential effectiveness of this approach.

## OXYTOCIN

Oxytocin is a neuropeptide produced primarily in the hypothalamic nuclei that acts in different areas and networks as a neurotransmitter and neuromodulator and appears to play a relevant role in social cognition-related processes.^151–153^ Therefore, intranasal oxytocin administration has been postulated as a potential treatment for social cognition deficits in different neurodevelopmental conditions, including SSD.^154^ A meta-analysis including 17 studies on different neurodevelopmental disorders, 8 of which included participants diagnosed with SSD, reported a significant small size effect on theory of mind (*g* = 0.21) and a non-significant moderate size effect on empathy (*g* = 0.49), and no significant effect on emotion recognition (*g* = 0.08). These effects did not appear to differ across diagnoses.^155^ A more recent trial that was not included in the meta-analysis, including 28 participants diagnosed with SSD, reported more negative findings.^156^ Considering these results, oxytocin-mediated neurotransmission and neuroregulation still appear as promising targets for the treatment of the social cognition component of CIAS, but intranasal oxytocin does not seem to represent an effective way to produce substantial positive results.^157,158^

## DISCUSSION

As CIAS represents one of the core components of SSD, with important repercussions on real-world functional outcomes and on the recovery process, its treatment currently represents a fundamental therapeutic target.^2,5,6^

Available antipsychotic treatment currently provides only small benefits as regards CIAS, but accurate management of antipsychotic medications is required to avoid a further worsening of cognitive performance: in this regard, avoiding prolonged treatments with first-generation molecules and high anticholinergic burdens represents a strategy that can produce significant positive effects. Avoiding long-term use and high dosages of anticholinergic molecules and benzodiazepines could also provide substantial benefits, particularly in patients that show significant CIAS.^14^

Already available molecules that are used in the treatment of other conditions, such as antidepressants, raloxifene, ondansetron, buspirone, and acetylcholinesterase inhibitors, do not appear to provide consistent positive effects, but some interesting and promising effects that require further validation have been observed with memantine.

As regards novel molecules that are currently under study with a primary antipsychotic effect, ulotaront did not appear to produce a significant effect in different Phase 3 trials, while xanomeline-trospium appears to provide both antipsychotic benefits and an independent pro-cognitive effect^132,134^; the latter, however, requires to be replicated in further studies.

As regards add-on treatments, d-serine and sodium benzoate did not provide fully satisfactory results, while novel molecules such a luvadaxistat and iclepertin in particular show promising results.^115,129^ The assessment of the cognitive effects of both luvadaxistat and iclepertin is currently underway in several different dedicated trials.

As regards potential interactions of novel molecules with currently available antipsychotic medication, add-on treatments such as luvadaxistat and iclepertin are being investigated in combination with the usual antipsychotic treatment of participants, so potential interaction with antipsychotic medication is already taken into account in their safety and tolerability profile. Xanomeline-trospium has been investigated as a stand-alone medication, and its potential interaction with antipsychotic medications is currently being assessed in the NCT05145413 trial.

Anti-inflammatory and antioxidant molecules, *N*-acetylcysteine and minocycline in particular could represent another viable strategy to treat CIAS, but available evidence on their effectiveness is currently too limited to provide clear treatment recommendations.^144–146,148^

While these promising molecules and novel approaches are currently being investigated, some treatment choices to improve CIAS in clinical contexts are available in the present moment: in particular, evidence-based cognition-oriented psychosocial interventions such as cognitive remediation^24,27,159,160^ and aerobic exercise^28–30^ reliably produce improvements in CIAS and can be currently implemented in rehabilitation settings, even in clinical contexts with limited available resources.^161–163^

Moreover, the cost of novel molecules for the treatment of CIAS and their accessibility in real-world clinical contexts remains to be investigated but might represent a noteworthy issue, particularly considering that income and ethnic inequalities represent a considerable issue in the treatment and the clinical management of schizophrenia both at a local and at a global level.^164–166^

An interesting perspective for future studies is the combination of pharmacological agents, be they already available or under development, with interventions that have an already-established effectiveness profile: this strategy has been currently attempted with iclepertin,^117^ and applying this approach also to other molecules could provide interesting results. The same holds true for the combination of novel molecules with other emerging treatments with preliminary evidence of effectiveness in CIAS, such as non-invasive brain stimulation.^167–170^

Another recommendation that should be reminded for future research is the need to implement in pharmacological trials an assessment CIAS that is validated, reliable and allows qualitative synthesis across studies: this could allow more powerful and more clear meta-analytical investigations and the development of straightforward clinical recommendations.^171^

In conclusion, current perspectives on pharmacological treatment of CIAS remain limited, and evidence-based psychosocial interventions still remain the most effective approach available in clinical practice, but several interesting pharmacological solutions are being developed and, if found to be reliably effective, could become available and game-changing options in the near future.
